# Predictors of Adherence in Three Low-Intensity Intervention Programs Applied by ICTs for Depression in Primary Care

**DOI:** 10.3390/ijerph18041774

**Published:** 2021-02-11

**Authors:** Adoración Castro, Azucena García-Palacios, Yolanda López-Del-Hoyo, Fermín Mayoral, María Ángeles Pérez-Ara, Rosa Mª Baños, Javier García-Campayo, María M. Hurtado, Cristina Botella, Alberto Barceló-Soler, Amelia Villena, Miquel Roca, Margalida Gili

**Affiliations:** 1 Research Institute of Health Sciences (IUNICS), University of Balearic Islands, 07122 Palma de Mallorca, Spain; m.perez@uib.es (M.Á.P.-A.); mroca@uib.es (M.R.); mgili@uib.es (M.G.); 2 Health Research Institute of the Balearic Islands (IdiSBa), 07120 Palma de Mallorca, Spain; 3 Department of Clinical and Basic Psychology and Biopsychology, Faculty of Health Sciences, University Jaume I, 12071 Castellón, Spain; azucena@uji.es (A.G.-P.); botella@uji.es (C.B.); 4 CIBER Physiopathology Obesity and Nutrition (CIBERobn), Carlos III Health Institute, 28029 Madrid, Spain; banos@uv.es; 5 Institute of Health Research of Aragon (IIS), Hospital Miguel Servet, 50009 Zaragoza, Spain; ylopez.iacs@gmail.com (Y.L.-D.-H.); jgarcamp@gmail.com (J.G.-C.); abarcelosoler@hotmail.com (A.B.-S.); 6 Primary Care Prevention and Health Promotion Research Network, RedIAPP, 28029 Madrid, Spain; 7 Department of Psychology and Sociology, University of Zaragoza, 50009 Zaragoza, Spain; 8 Mental Health Department, Institute of Biomedicine of Malaga, University Regional Hospital of Malaga, 29010 Málaga, Spain; fermin.mayoral.sspa@juntadeandalucia.es (F.M.); marienahurtado@gmail.com (M.M.H.); amelia.villena.j@gmail.com (A.V.); 9 Department of Psychological, Personality, Evaluation and Treatment, University of Valencia, 46010 Valencia, Spain

**Keywords:** depression, primary care, ICTs, adherence, predictors

## Abstract

Depression is one of the most common disorders in psychiatric and primary care settings, and is associated with disability, loss in quality of life, and economic costs. Internet-based psychological interventions have been shown to be effective in depression treatment but present problems with a low degree of adherence. The main aim of this study is to analyze the adherence predictors in three low-intensity interventions programs applied by Information and Communication Technologies (ICTs) for depression. A multi-center, randomized, controlled clinical trial was conducted with 164 participants with depression, who were allocated to: Healthy Lifestyle Program, Positive Affect Promotion Program or Mindfulness Program. Sociodemographic characteristics, Patient Health Questionnaire-9, Visual Analog Scale, Short Form Health Survey, Positive and Negative Affect Schedule, Five Facets Mindfulness Questionnaire, Pemberton Happiness Index and Treatment Expectancy Questionnaire were used to study adherence. Results showed that positive affect resulted in a predictor variable for Healthy Lifestyle Program and Positive Affect Promotion Program. Perceived health was also a negative adherence predictor for the Positive Affect Promotion Program. Our findings demonstrate that there are differences in clinical variables between treatment completers and non-completers and we provide adherence predictors in two intervention groups. Although new additional predictors have been examined, further research is essential in order to improve tailored interventions and increase adherence treatment.

## 1. Introduction

About 30 million Europeans are estimated to suffer from depression [1]. In Spain, prevalence of depression ranges between 13.9% and 29% [2,3] and more than 50% of depressive patients are attended in primary care [4]. It is well established that pharmacotherapy and psychotherapy, or a combination of both, are the best option for treating depression [5,6]. Nevertheless, only 65% of patients show good medication adherence [7] and face-to-face psychotherapy meetings present difficulties in delivering intervention given the face-to-face time required, the cost, and the lack of trained professionals [8,9,10,11]. Consequently, Internet-based treatments may help to overcome these impediments making it possible to deliver therapy to a large number of people in their own environment, at a convenient moment [12,13].

A large body of research has reported that Internet-based intervention programs have been shown as an efficacious therapeutic option for the treatment of many mental health problems including depression [14,15,16,17,18,19]. Despite its background, Internet interventions present problems with a high degree of non-adherence, with many participants not completing the whole intervention [20,21]. Dropout during treatment ranged from 0 to 78% in Internet-based interventions for psychological disorders [22]. Regarding Internet-based programs for depression, the literature shows that adherence tends to be higher when the intervention has some guidance by a professional, with average levels of adherence estimated at 72%. By contrast, adherence rate is around 25% when the intervention is unguided [15]. With regard to the length of the interventions, Christensen et al. [23] found that longer programs are associated with higher rates of dropout.

A large body of research has focused on the study of adherence predictors in the past years [21,24,25,26,27,28,29,30,31]. Nevertheless, the results are contradictory. Previous studies have shown that specific sociodemographic characteristics, such as gender and age, are related to adherence treatment. In particular, it has been shown that females [24,26] and younger individuals are more likely to adhere to online interventions [21,24,25]. But opposite findings have also been reported. Several studies have found that better adherence was associated with older individuals [26,29,32,33] and with males [28,33].

In reference to clinical predictors, baseline depression severity is one of the most commonly explored characteristics. Many studies have reported that lower levels of baseline depression predicted adherence [21,25,28] whereas others studies found the opposite result [24,27] or even no relationship [29].

Other psychological characteristics were also studied, such as pre-treatment expectations, perceived credibility of the treatment, and/or attitudes toward Internet-interventions. Nevertheless, results showed that these variables were not related with treatment completion [27,34]. It is important to note that these results must be treated with caution because of the heterogeneity of the studies in terms of the differences of sample size, intervention characteristics, and adherence definitions.

For all these reasons, it is crucial to examine new additional characteristics such as factors of mindfulness, positive and negative affect, and well-being, in order to have new knowledge of which variables are associated and predict treatment adherence.

The aim of the present study is to examine the differences in sociodemographic and clinical characteristics between treatment completers and non-completers and to analyze the adherence predictors in three low-intensity intervention programs applied by ICTs: Healthy Lifestyle Program; Positive Affect Promotion Program and Mindfulness Program, for depression treatment in Spanish Primary Care.

## 2. Materials and Methods

### 2.1. Study Design

The current study is a secondary analysis of a multi-center, controlled, randomized clinical trial conducted with patients with depression recruited in the primary care. They were randomized to receive one of four intervention groups: (a) improved primary care usual treatment (iTAU), (b) Healthy Lifestyle Program + iTAU, (c) Positive Affect Promotion Program + iTAU or (d) Mindfulness Program + iTAU.

Trial Number Registration from the original study is ISRCTN82388279. The aim of the original study was to assess the effectiveness of three low-intensity intervention programs applied by ICTs (Healthy Lifestyle Program, Positive Affect Promotion Program and Mindfulness Program) compared with a control condition. The research protocol and main results have been reported elsewhere [35,36].

### 2.2. Participants

In the original study, a total of 221 patients with depression were recruited in primary care settings, between March 2015 and March 2016 in the Spanish regions of Aragon, Andalucía and Baleares. Inclusion criteria were: older than 18 years old; DSM-5 diagnose of Major Depression or Dysthymia, with mild or moderate severity depression assessed as score lower than 14 according to the Patient Health Questionnaire (PHQ-9) [37], with symptoms presented for at least two months, be able to read and understand the Spanish language, and have the capacity to understand and sign the written informed consent form. We excluded patients who were diagnosed with any disease that may affect central nervous system (brain pathology, traumatic brain injury, dementia), or any psychiatric disorder other than major depression, dysthymia, anxiety disorders, or personality disorders, any medical, infectious or degenerative disease that may affect mood; patients with delusional ideas or hallucinations consistent or not with mood and with suicide risk.

For the present study, a total of 164 participants were selected and included, those who were allocated to one of the intervention groups (Healthy Lifestyle Program; Positive Affect Promotion Program and Mindfulness Program). Those allocated to iTAU were excluded from the analysis because they were control group, with no access to online treatment. Our small sample size (*n* = 164) led to a lack of statistical power, resulting in our major limitation.

### 2.3. Intervention Groups Description

All interventions consisted of one face-to-face group session and four self-guided online therapeutic modules.

Face-to-face session involved up to five participants of the same intervention program and was 90 min in length. The aim of this session was to explain the program structure and main components of treatment, clarify the instructions for the use of the online platform, and to motivate participants for change, as well as reinforce commitment and treatment adherence. The participants were given a leaflet with a brief summary of the topics covered in this session.

Online modules were a low-intensity psychological intervention oriented to work on different psychological techniques. The duration of each module was approximately 60 min. Modules included an explanation of the module contents and exercises to practice. These modules were sequential, in order to move step by step, all along the program, with the possibility of reviewing module contents once they were finished. Duration of each intervention program varied among users, but it is estimated that for the most people, it lasted between 4 and 8 weeks (around one module for a week).

The Healthy Lifestyle Program provides information to understand the relationship between physical and mental health and physical activity, diet and sleep control. Modules are: (1) Beginning of a lifestyle change, focused on the importance of healthy lifestyle to improve emotional health and general well-being; (2) physical activity—learning to move, to give information about exercises to improve mood, to increase motivation, to start being more active, and to maintain this physical activity; (3) diet—learning to eat, focused on the importance of diet to achieve a good physical and mental health, and the role of the Mediterranean diet in the prevention and treatment of depression; and (4) sleep—the importance of good sleep, to understand the relationship between sleep, and general health.

The Positive Affect Promotion Program aims to decrease depressive symptomatology and to prevent relapses using the promotion of well-being and positive affect. The modules are: (1) Learning to live, focused on the importance of establishing and maintaining an adequate activity level and the relevance of choosing activities that are significant for the individual; (2) learning to enjoy, to give education about the effect of positive emotions and to train the patient in learning procedures to increase the likelihood of experiencing positive emotions; (3) accepting to life, focused on the training to the patient in concentrating on positive emotions related with the past or the future; and (4) living and learning, to train the patient in understanding life as a continuous process of learning and personal growth, emphasizing the training in strategies to promote psychological strengths, resilience, and meaningful goals linked to important values.

The Mindfulness Program has the objective to decrease depressive symptomatology using mindfulness, emphasizing the benefits of its therapy. The modules are: (1) Getting to know mindfulness, to explain what mindfulness is, prejudices about it, the inattention problem, and some of its main benefits and recommendations to practice it; (2) establishing formal and informal practices, focused on the importance of the establishment of formal and informal practice; (3) through management, body scan practice and values, to help people to see the importance of values to keep a regular mindfulness practice; and (4) Self-compassion. Integrating mindfulness in everyday life, to establish a regular practice of mindfulness to be indefinitely kept.

Full details of the interventions are described in Castro et al. [35] and Gili et al. [36].

### 2.4. Adherence Definition

In the context of this analysis, adherence refers to the extent to which individuals complete the whole intervention in the post-test evaluation. Therefore, a participant was considered to be part of the adherence group (completer participant) if they completed the whole intervention (1 face-to-face session plus 4 self-guided online therapeutic modules) at the post-test assessment. A participant who did not complete the whole intervention at the post-test was considered non-completer participant.

### 2.5. Instruments

Case report form (CRF). The patient’s sociodemographic and clinical characteristics were gathered using a CRF: gender, date of birth and age, place of residence, family status, if they live alone or accompanied, level of education, work status and income level. Psychopharmacologic medication (yes vs. no) and the number of physician visits in the last 12 months were also collected.

The Mini International Neuropsychiatric Interview 5.0 (M.I.N.I. 5.0) [38]. This diagnostic interview was used at the screening to assess current depression and comorbid disorders. This measure is a structured diagnostic interview based on the DSM-IV and the International Classification of Diseases-10 criteria. The Spanish version was used for this study [39].

Patient Health Questionnaire-9 (PHQ-9) [40]. This instrument was used to evaluate depression severity. Participants describe their mood of the last two weeks prior to evaluation, with items ranging from 0 to 3 denoting “not at all,” “several days,” “more than half the days,” and “nearly every day,” respectively. Total scores range from 0 to 27. The Spanish version was used and has been shown to have good psychometric properties (for the diagnosis of any disorder, k = 0.74; overall accuracy, 88%; sensitivity, 87%; specificity, 88%) [37].

The Visual Analog Scale of the EuroQol (VAS); [41]. This scale was used to assess the respondent’s self-rated health. The VAS is a 10-cm vertical line with the best and worst possible health states score 100 and zero, respectively. Spanish version was used in this study [42].

The Short Form Health survey (SF-12v1) [43]. This scale was used as a measure of health-related quality of life from the patient’s perspective. The SF-12 scoring algorithm yields a physical component scale (general health, bodily pain, role-physical, physical functioning) and a mental component scale (mental health, role-emotional, social-functioning, vitality). Both subscales were used as continuous variables using Spanish norms [44]. The Spanish version was used in this study and presents good psychometrics properties (Cronbach α = 0.7) [44].

Positive and Negative Affect Schedule (PANAS) [45]. This scale was used to evaluate the positive and negative affect. It consists of 20 items that evaluate two independent dimensions: Positive Affect (PA) (10 items) and Negative affect (NA) (10 items). Total score for each subscale ranges from 5 to 50, using a 5-point Likert-type scale (1 = very slightly or not at all, 5 = very much). “Trait” version was used in this study. The Spanish version showed adequate internal consistency (Cronbach’s α between 0.87 and 0.91) [46].

Five Facets Mindfulness Questionnaire (FFMQ) [47]. This questionnaire comprises 39 items, rated on a 5-point Likert-type scale (1 = never or very rarely true, 5 = very often or always true), and was used to assess five facets or factors of mindfulness: observing (tendency to attend to internal and external experiences such as sensations, emotions, sounds), describing (tendency to describe and label these experiences with words), acting with awareness (tendency to give full awareness and attention to current activities or experiences), not judging inner experience (tendency for a nonevaluative stance toward inner experiences), and not reacting to inner experience (tendency to allow thoughts and feelings to come and go, without getting caught up in them). The Spanish version was used in this study and has shown good internal consistency (Cronbach’s α > 0.80) [48].

The Pemberton Happiness Index (PHI) [49]. This index consists of 11 items related to different domains of remembered well-being, each with an 11-point Likert-type scale (0 = fully disagree, 10 = fully agree), and 10 items related to experienced well-being, with dichotomous response options (yes/no). The remembered well-being score is calculated with the mean score of the 11 items and varies from 0 to 10. The items for experienced well-being are transformed into a single score from 0 (zero positive experiences and 5 negative experiences) to 10 (5 positive experiences and no negative experiences). To calculate the overall PHI index, which included remembered and experienced well-being, individuals’ scores of the 11 items related to remembered well-being plus the sum of scores on the experienced well-being were summed; the total sum is then divided by 12, so the resulting PHI total mean score also ranges from 0 to 10. The validated Spanish version showed adequate psychometric properties (Cronbach’s α > 0.80) [49].

Treatment Expectancy Questionnaire [50]. This questionnaire was used to assess the patient’s expectations about the treatment that he/she is going to receive. It consists of 6 items scored between 0 (nothing) to 10 (very much). Total score is the sum of each item and the maximum rate is 60. Higher scores mean better expectations about the treatment that he/she is going to receive.

### 2.6. Procedure

Patients were recruited in Spanish Primary Care settings. When the general practitioner (GP) identified a potential participant during a visit, he or she explained to the participant the characteristics of the study. If the participant was interested on it, he or she signed an informed consent form and the GP filled a referral form describing the sociodemographic characteristics of the patient and a checklist for inclusion and exclusion criteria. GP also gave him or her the patient’s information sheet and a handout describing the study. Then, the GP sent these documents to the local researcher. Participants were interviewed after a few days by the researcher, which administered psychological assessment instruments related with inclusion and exclusion criteria by phone. All participants fulfilled their assessments through a web (https://psicologiaytecnologia.labpsitec.es/). Included participants were randomized to 1 of the 4 groups by an independent researcher using a computer-generated random number. The Ethical Review Board of the regional health authority approved the study (Ref: IB 2144/13 PI). More details about the study design, recruitment, randomization and data collection procedure are described elsewhere [35,36].

### 2.7. Outcomes and Data Analyses

Three intervention groups of participants were selected regarding the assigned low-intensity psychological intervention applied by ICTs (Healthy Lifestyle Program vs. Mindfulness Program vs. Positive Affect Promotion Program). Descriptive analysis examined the differences between groups of adherence (treatment completer vs. non-completer) for each intervention group and was computed in terms of mean and standard deviation for continuous variables, and frequency with percentage for ordinal and nominal variables. Discrete variables were compared using the chi-square test and Student t tests were used for continuous variables comparisons. A post-hoc analysis was conducted in order to explore the intervention group differences regarding adherence considering adherence as a dichotomous variable (completers vs. non-completers) through chi-square homogeneity test, and as a continuous variable (number of modules completed) by ANOVA test. Logistic regression models using adherence as a dependent variable were constructed using independent predictor variables. Subsequent binary logistic regression analyses for each intervention group were conducted to build parsimonious models. Significance associated probability for analyzed contrasts were fit to 0.05, while significance analyses for b coefficients in logistic regressions and for Odd Ratios were calculated based on 95% CI. The effects of b coefficients of final logistic regressions were bootstrapped with 1000 replications. The analyses were performed using IBM SPSS v22 (IBM Corp, Armonk, NY, USA).

## 3. Results

### 3.1. Sample Description

Final analyses were undertaken on 164 subjects, those who were allocated in one of the three intervention groups, with 32.9% receiving Healthy Lifestyle Program, 32.9% receiving Mindfulness Program, and 34.1% receiving Positive Affect Promotion Program. The sample was predominantly female (79.9%) and the mean age was 45.03 (SD = 11.16) years. Most were married (58%), lived accompanied (83.4%), had primary/secondary educational level (68%) and had no paid employment (51.6%), and had an income level predominantly more than 2 NMW (35.6%). With respect to clinical variables, the PHQ-9 mean of the total sample was 12.62 (SD = 2.34) and 70.9% used psychopharmacologic treatment.

Regarding differences in sociodemographic and clinical characteristics between treatment completers and non-completers in each intervention groups, analysis found that the Healthy Lifestyle Program group had statistically significant differences in Positive Affect baseline scores, self-measured by the Positive Affect dimension from PANAS Schedule (PA-PANAS) (mean = 23.32 vs. mean = 16.25; *p* = 0.000), baseline scores from “Describing” FFMQ Subscale (mean = 26.50 vs. mean = 22.13; *p* = 0.022) and baseline global average PHI (mean = 5.26 vs. mean = 3.87; *p* = 0.007). In the Positive Affect Promotion Program, statistically significant differences between treatment completers and non-completers were found in baseline perceived health, self-measured by the VAS (mean = 39.44 vs. mean = 56.82; *p* = 0.003) and baseline scores from “Not reacting to inner experience” FFMQ Subscale (mean = 19.22 vs. mean = 15.68; *p* = 0.013). In the Mindfulness Program, statistically significant differences between treatment completers and non-completers were not found in sociodemographic and clinical variables.

The sample group’s sociodemographic characteristics and comparisons between both groups are shown in Table 1.

Results of the post-hoc analysis found neither statistically significant differences in the proportions of completers and non-completers between intervention groups (χ^2^_(2)_ = 4.417; *p* = 0.110), nor in terms of modules completed (mean = 3.01 in Healthy Lifestyle; mean= 3.37 in Mindfulness Program and mean = 2.70 in Positive Affect Promotion Program; F = 1.608; *p* = 0.204).

### 3.2. Logistic Regression Analysis Results

A logistic regression was carried out to test if there were interactions between the predictors variables. Because of an effect of interaction between some predictor variables and intervention variable, models were analyzed separately. After that, a bivariate analysis was performed between independent baseline demographic and clinical variables and adherence (completers vs. non-completers) as a dependent variable in order to identify the potential predictors. No reactivity to inner experience FFMQ subscale and positive affect (PA-PANAS) variables were significantly associated (*p* < 0.10). After a literature review, models were also adjusted by the following variables: age, sex, educational level, depression symptomatology (PHQ-9), perceived health (VAS), and treatment expectations.

Odds ratio (OR) and 95% confidence intervals for each adherence predictor in each intervention group are shown in Table 2. Positive affect (self-measured by the PA-PANAS) was a significant adherence predictor (*p* = 0.005) for Healthy Lifestyle Program and perceived health (self-measured by the VAS) for Positive Affect Promotion Program (*p* = 0.012). Models were significant fit of data for Healthy Lifestyle Program (X^2^_(8)_ = 23, *p* = 0.003) and for Positive Affect Promotion Program (X^2^_(8)_ = 21.628, *p* = 0.006) but not for Mindfulness Program (X^2^_(8)_ = 2.479, *p* = 0.963).

**Figure 1:**
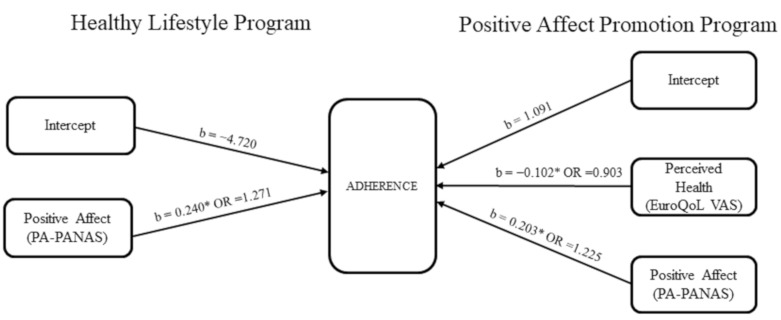
Regression models for Healthy Lifestyle program and Positive Affect Promotion Program. Notes: Healthy Lifestyle model: R^2^ = 0.25 (Hosmer & Lemeshow), R^2^ = 0.295 (Cox & Snell), R^2^ = 0.394 (Nagelkerke). X^2^_(1)_ = 16.11, *p* < 0.001; Positive Affect Promotion Model: R^2^ = 0.29 (Hosmer &Lemeshow), R^2^ = 0.336 (Cox & Snell), R^2^ = 0.449 (Nagelkerke). X^2^_(1)_ = 16.358, *p* < 0.001. b: Regression coefficient; OR: Odds ratio; * *p* < 0.05 (based on 95% bootstrap confidence intervals based in 1000 samples).

The most parsimonious models to predict adherence, based on our sample and predictors, are shown in Table 3. Positive affect, self-measured with the PA-PANAS, was a significant predictor for both Healthy Lifestyle Program and Positive Affect Promotion Program. Specifically, 1-point increase was associated with 27% greater odds of adhering to the Healthy Lifestyle Program, while 1-point increase in the Positive Affect Promotion Program group was associated with 22.5% greater odds of adherence. Perceived health, self-measured with the EuroQoL VAS scale, was a significant negative predictor of adherence for the Positive Affect Promotion Program: 1-point decrease in this scale was associated with 10% greater odds of adhering to this intervention. These models were significant fits of data: χ^2^_(1)_ = 16.11, *p* < 0.001, for Healthy Lifestyle Program; χ^2^_(2)_ = 16.358, *p* < 0.001 for the Positive Affect Promotion Program (Figure 1) and correctly classified 78.3% of participants in the Healthy Lifestyle Program and 77.5% in the Positive Affect Promotion Program.

For Mindfulness Program, the most parsimonious model was found, although it was not significant fit of data (χ^2^_(1)_ = 3.422, *p* = 0.181).

## 4. Discussion

Our main objectives were to examine the sociodemographic and clinical differences between treatment completers and non-completers and to analyze the adherence predictors in three low-intensity intervention programs applied by ICTs (Healthy Lifestyle Program, Mindfulness Program and Positive Affect Promotion Program) for depression in primary care.

Our completion treatment rates were relatively low in the whole sample (41.5%) and in each intervention groups with no differences between them (40.7% in Healthy Lifestyle Program; 51.8% in Mindfulness Program and 32.1% in Positive Affect Promotion Program). Our results were comparable to previous research that showed that only about half of the participants of unguided interventions complete all modules [51] and nearly around 60% of participants dropout from the intervention [15]. However, adherence rates reported herein are higher than those reported in previous studies, in which they do not exceed 20% of participants who completed all treatment sessions [26,52,53]. In our study just one intervention group (Mindfulness Program) presented more treatment completers than the non-completers (even the percentage is quite similar) and treatment adherence rate is in line with a previous online mindfulness-enhanced Cognitive Behavior Therapy pilot trial for depression, where a 59.1% of completion rate was found [54].

Contrary to prior findings, sociodemographic characteristics were not associated to adherence condition in our sample. This is inconsistent with the findings of earlier researches on this subject which found that participant’s characteristics, such as age, gender, or educational level are associated with adherence [24,29]. In terms of clinical variables, some were found to be associated to adherence condition in our sample. In particular, comparisons between treatment completers and non-completers in Healthy Lifestyle Program suggested that higher basal levels of positive affect, self-measured by the PA-PANAS, higher basal scores in the FFMQ Subscale Describing and higher basal levels of well-being, self-measured by the PHI, were associated with adherence condition. Regarding Positive Affect Promotion Program, adherence condition was associated with lower basal levels of perceived health, self-measured with VAS, and higher basal scores in the FFMQ Subscale Not reacting to inner experience. These findings cannot be comparable with previous literature because, to our knowledge, this is the first study which examines these clinical characteristics on adherence.

When logistic regression analyses were performed, some of our findings were attenuated. According to adherence predictors, a consistent finding was found: for patients in Healthy Lifestyle Program and Positive Affect Promotion Program, positive affect, self-measured by the PA-PANAS, predicted adherence. Hence, those participants that presented high positive affect scores at baseline tend to adhere more than those with low positive affect levels. A possible explanation of this finding could be that positive affect assesses personal traits such as interest, excitement, enthusiasm, among others. These traits could be related to the motivation to start and use a non-conventional psychotherapy such as an intervention program applied by ICTs, as the Healthy Lifestyle Program or the Positive Affect Promotion Program are. Also, it is important to note that Positive Affect Promotion Program is mainly designed to decrease depressive symptomatology through the promotion of positive affect and subjective-well-being, and this could maybe explain why people with higher basal levels of positive affect persist in this program. Nevertheless, our finding cannot be compared with the previous literature. To the best of our knowledge, this predictor has not been explored before. Therefore, more research is required to determine the impact of this predictor on adherence in other intervention programs applied by ICTs for depression.

Perceived health, self-measured with the EuroQoL VAS, also predicted adherence but just in Positive Affect Promotion Program. Therefore, those participants that perceived their health to be worse at baseline assessment are more likely to adhere to the treatment. As we reported before, Positive Affect Promotion Program is also designed to promote well-being, and well-being could be related with perceived health. Participants with worse perceived health could feel the treatment useful for their health and maybe they are more like to keep on this program.

The result that perceived health is related to adherence is partially in line with the findings of an earlier study on this subject, where Castro et al. [29] found that in a self-guided Internet-based intervention, perceived health also predicted adherence, but only when depressive symptomatology improved. This finding is partially in line with those studies which showed that those patients with worst clinical status at baseline are more likely to keep using the intervention: Batterham et al. [24] found that better adherence was predicted by higher depression severity, higher anxiety severity, and a greater level of dysfunctional thinking. Furthermore, Fuhr et al. [27] found that higher depressive symptoms predicted higher adherence levels.

In fact, a growing body of research found that low depression severity at baseline predicted higher adherence [21,25,26,28]. Contrary to prior studies, findings from the present study suggest that the baseline severity of depression does not significantly predict adherence treatment.

No adherence predictors were found for Mindfulness program. Possible explanations of this fact could be that the percentage of treatment completers and non-completers are almost equal. Another potential explanation could be that this study examined a specific number of predictors of adherence, and perhaps other possible variables that could predict adherence treatment have not been included in this study.

There are several limitations associated with this research. First, our sample size is smaller than the previous studies [24,27] and considerable baseline missing data were found in some measurements, leading to a lack of statistical power, being able to consider this study as a pilot study. A replication of these analyses in a larger sample is recommended in order to provide firm conclusions. Second, although an objective measure of adherence was used in this study (percentage of patients who completed the whole intervention), the defining criterion was exhaustive. Adherence definitions vary across studies and these criteria do not necessarily correlate strongly. Third, it was not possible to find the significant fit of data model for the Mindfulness Program. Finally, the majority of the sample was female and depressed and dysthymic patients were not analyzed separately. For these reasons, results could be interpreted with caution. Despite these limitations, our study has several strengths: to the best of our knowledge, this is the first study analyzing adherence predictors in three low intensity psychological treatments applied by ICTs on depression in Spanish Primary Health care. Second, additional clinical variables, such as positive and negative affect, well-being, mindfulness and treatment expectancy, were also proposed and examined as possible characteristics associated to adherence. Finally, we presented the most parsimonious models for our sample and data in two of the three intervention conditions.

Although the adherence rates found in this research are low in the three intervention groups, it is surprising that only about 30% of the participants completed the Positive Affect Promotion Program. More specific research is needed to understand and improve the adherence in this type of intervention in particular, and Internet interventions in general. It is well-known that adherence is an important measure of acceptability, appropriateness, and effect of psychological treatment [55]. The identification of adherence predictors allows us to identify which populations would most benefit from this intervention and also to take into account all these predictors for future development of Internet interventions for depression in order to optimize it. As Karyotaki et al. [26] explained “it is important to find out what works best for whom.” Thus, this knowledge will help in using Internet interventions programs in the most efficient and effective way [26]. Nevertheless, it is important to keep examining additional potential predictors, such as personality styles, motivation and preferences, chronicity, and/or comorbidities, in future studies in order to adapt and increase adherence to Internet intervention programs for depression.

## 5. Conclusions

Our research found that there are some clinical variables associated with adherence in two low-intensity intervention programs applied by ICTs. In particular, treatment completers present higher basal levels of positive affect, higher basal scores in the FFMQ Subscale Describing and higher basal levels of well-being compared to treatment non-completers in Healthy Lifestyle Program. In Positive Affect Promotion Program, treatment completers present lower basal levels of perceived health and higher basal scores in the FFMQ Subscale Not reacting to inner experience. With regard to adherence predictors, for patients in Healthy Lifestyle Program and Positive Affect Promotion Program, positive affect predicts adherence. That is, those participants that present high positive affect scores at baseline tend to adhere more than those with low positive affect level. Finally, perceived health, is a negative predictor of adherence but just in Positive Affect Promotion Program; those participants that perceived their health to be worse at baseline assessment are more likely to adhere to the treatment. Significant differences in sociodemographic and clinical characteristics between treatment completers and non-completers were not found nor adherence predictors in Mindfulness Program.

## Figures and Tables

**Table 1:** Baseline sociographic and clinical characteristics and comparisons between treatment completers and non-completers.

		Healthy Lifestyle Program (*n* = 54)		Mindfulness Program (*n* = 54)		Positive Affect Promotion Program (*n* = 56)	
Sociodemographic and Clinical Variables	Total (*n* = 164; 100%)	Completers (*n* = 22; 40.75%)	Non-Completers (*n* = 32; 59.25%)	Completers (*n* = 28; 51.85%)	Non-Completers (*n* = 26; 48.5%)	Completers (*n* = 18; 32.14%)	Non-Completers (*n* = 38; 67.85%)
Age, mean (SD)	45.03 (11.16)	41.86 (10.21)	45.69 (9.8)	48.89 (12.80)	44.65 (12.78)	48.06 (8.83)	42.29 (10.64)
Sex, *n* (%)	164						
Male	33 (20.1)	6 (27.3)	8 (25)	4 (14.3)	3 (11.5)	6 (33.3)	6 (15.8)
Female	131 (79.9)	16 (72.7)	24 (75)	24 (85.7)	23 (88.5)	12 (66.7)	32 (84.2)
Family Status, *n*(%)	157						
Single	66 (42)	11 (50)	15 (51.7)	11 (39.3)	9 (40.9)	6 (33.3)	14 (36.8)
Married	91 (58)	11 (50)	14 (48.3)	17 (60.7)	13 (59.1)	12 (66.7)	24 (63.2)
Educational Level, *n* (%)	153						
Primary/Secondary	104 (68)	15 (71.4)	24 (82.8)	15 (57.7)	13 (59.1)	13 (72.2)	24 (64.9)
University	49 (32)	6 (28.6)	5 (17.2)	11 (42.3)	9 (40.9)	5 (27.8)	13 (35.1)
Lives, *n* (%)	157						
Alone	26 (16.6)	5 (22.7)	5 (17.2)	6 (21.4)	5 (22.7)	1 (5.6)	4 (10.5)
Accompanied	131 (83.4)	17 (77.3)	24 (82.8)	22 (78.6)	17 (77.3)	17 (94.4)	34 (89.5)
Work Status, *n* (%)	155						
Unemployment	80 (51.6)	11 (52.4)	15 (51.7)	18 (66.7)	10 (43.5)	8 (44.4)	18 (48.6)
Paid employment	75 (48.4)	10 (47.6)	14 (48.3)	9 (33.3)	13 (56.5)	10 (55.6)	19 (51.4)
Income level, *n* (%)	149						
<NMW ^a^	48 (32.2)	7 (35)	9 (31)	8 (29.6)	7 (33.3)	5 (29.4)	12 (34.4)
1–2 NMW	48 (32.2)	7 (35)	11 (37.9)	7 (25.9)	6 (28.6)	7 (41.2)	10 (28.6)
>2 NMW	53 (35.6)	6 (30)	9 (31)	12 (44.4)	8 (38.1)	5 (29.4)	13 (37.1)
Depression severity							
PHQ ^b^ Basal, mean (SD)	12.62 (2.34)	11.86 (2.7)	13.06 (2.2)	12.82 (2.19)	12.50 (2.93)	12.39 (1.97)	12.74 (2.07)
Change in depression severity, *n*	106						
PHQ-Change ^c^, mean, SD	3.22 (5.93)	4.67 (4.33)	3.38 (4.96)	2.96 (6.82)	5.73 (6.96)	1.11 (4.56)	1.92 (7.63)
Perceived Health, *n*	132						
EuroQoL VAS ^d^, mean (SD)	50.08 (22.44)	55 (24.6)	49.57 (18.46)	47.86 (24.85)	50.56 (28.38)	39.44 (18.30) **	56.82 (16.15) **
Physical Health SF-12 ^e^, mean(SD)	43.23 (11.26)	44.06 (9.18)	41.68 (9.31)	44.71 (14.32)	46.37 (13.65)	38.37 (6.67)	43.64 (11.25)
Mental Health SF-12, mean (SD)	27.07 (10.12)	28.56 (12.34)	27.41 (10.57)	25.36 (10.28)	27.07 (9.85)	29.23 (10)	25.61 (7.53)
PANAS ^f^, *n*	132						
Positive Affect, mean (SD)	18.70 (6.22)	23.32 (6.85) ***	16.25 (3.88) ***	17.50 (4.89)	19.50 (7)	19.33 (6.94)	17.14 (5.78)
Negative Affect, mean (SD)	28.58 (8.29)	27.77 (7.93)	28.29 (8.13)	28.61 (7.94)	32 (9.6)	27 (8.07)	28.18 (8.95)
FFMQ ^g^, *n*	131						
Observing Subscale, mean (SD)	20.69 (5.52)	21.05 (5.09)	20.17 (5.65)	21.46 (6.8)	21.50 (4.3)	20.22 (4.58)	19.59 (5.95)
Describing Subscale, mean (SD)	23.43 (7.06)	26.5 (5.72) *	22.13 (6.59) *	22.61 (7.62)	24.06 (7.51)	23.28 (7.08)	22.36 (7.44)
Acting with awareness Subscale, mean (SD)	23.09 (6.11)	23 (7.38)	22.96 (5.64)	23.82 (5.96)	22.61 (6.83)	22.56 (4.42)	23.23 (6.53)
Not judging inner experience Subscale, mean (SD)	21.55 (6.92)	22.27 (6.92)	21.78 (6.26)	20.82 (8.67)	19.83 (6.26)	20.56 (5.26)	23.73 (6.89)
Not reacting to inner experience Subscale, mean (SD)	18.40 (5.15)	20.64 (5.76)	18.13 (4.94)	18.43 (4.96)	18.50 (5.84)	19.22 (4.27) *	15.68 (4.22) *
Global Average PHI ^h^, *n*, mean (SD)	128						
	4.36 (1.83)	5.26 (1.82) **	3.87 (1.39) **	4.17 (1.66)	4.12 (2.34)	4.20 (1.85)	4.52 (1.83)
Physician visits, *n*, mean (SD)	133						
	6.63 (8.89)	4.55 (5.76)	6.36 (10.19)	6.46 (7.67)	5.47 (5.33)	5.94 (6.88)	9.96 (13.12)
Psychopharmacologic treatment, *n* (%)	127						
No	37 (29.1)	9 (47.4)	5 (23.8)	10 (35.7)	4 (25)	3 (17.6)	6 (23.1)
Yes	90(70.9)	10 (52.6)	16 (76.2)	18 (64.3)	12 (75)	14 (82.4)	20 (76.9)
Treatment expectations, *n*, mean (SD)	128						
	44.07 (9.54)	44.82 (8.59)	44.32 (8.75)	41.39 (13.34)	44.82 (8.41)	46.22 (7.21)	44.14 (8)
Adherence, *n* (%)							
Completers	68 (41.5)						
No-Completers	96 (58.5)						

Notes: ^a^ National Minimum Wage; ^b^ Patient Health Questionnaire 9 items; ^c^ Differences in PHQ-9 at baseline assessment minus PHQ-9 scores at post-treatment assessment; ^d^ Visual Analog Scale of the EuroQoL; ^e^ Short Form Health Survey; ^f^ Positive and Negative Affect Schedule; ^g^ Five Facets Mindfulness Questionnaire; ^h^ The Pemberton Happiness Index; * *p* < 0.05; ** *p* < 0.01; *** *p* < 0.001.

**Table 2:** Binary logistic regression for Healthy Lifestyle Program, Mindfulness Program, and Positive Affect Promotion Program.

	Healthy Lifestyle Program				Mindfulness Program				Positive Affect Promotion Program			
			95% CI ^a^				95% CI				95% CI	
	OR ^b^	*p* ^c^	Lower	Upper	OR	*p*	Lower	Upper	OR	*p*	Lower	Upper
Sex	0.176	0.148	0.017	1.851	0.788	0.828	0.092	6.775	0.362	0.426	0.030	4.418
Age	0.981	0.704	0.891	1.081	1.029	0.353	0.969	1.092	1.083	0.155	0.970	1.208
Educational Level	2.411	0.396	0.316	18.379	0.875	0.863	0.193	3.960	0.421	0.422	0.051	3.483
PHQ-9 ^d^	1.084	0.685	0.736	1.597	1.036	0.791	0.797	1.348	0.598	0.112	0.317	1.127
EuroQoL VAS ^e^	0.968	0.293	0.912	1.028	1.010	0.537	0.979	1.042	0.869 *	0.012	0.778	0.970
Positive Affect (PA-PANAS) ^f^	1.456 *	0.005	1.120	1.894	0.914	0.274	0.777	1.074	1.186	0.103	0.966	1.457
No reactivity to inner experience subscale FFMQ ^g^	1.111	0.278	0.918	1.345	1.028	0.729	0.879	1.202	0.965	0.785	0.749	1.244
Treatment expectations	0.985	0.791	0.883	1.099	0.992	0.854	0.911	1.080	1.050	0.456	0.924	1.194

Notes: ^a^ Confidence interval; ^b^ Odds Ratio; ^c^ Associated Probability; ^d^ Patient Health Questionnaire 9 items; ^e^ Visual Analog Scale of the EuroQoL; ^f^ Positive dimension of the Positive and Negative Affect Schedule; ^g^ Five Facets Mindfulness Questionnaire; * *p* < 0.05.

**Table 3:** Coefficients of the models (Model 1: Healthy Lifestyle Program; Model 2: Mindfulness Program; Model 3: Positive Affect Promotion Program) predicting whether a patient was adhered to the intervention.

		B ^a,b^	95% CI ^c^ for Odds Ratio		
			Lower	Odds ^d^	Upper
Healthy Lifestyle Program	Intercept	−4.720 [−11.252, −2.575]			
	Positive Affect (PA-PANAS) ^e^	0.240 * [0.134, 0.556]	1.092	1.271	1.479
Mindfulness Program	Intercept	1.66 [−3.578, 3.566]			
	Age	0.039 [−0.018, 0.130]	0.985	1.040	1.098
	Positive Affect (PA-PANAS)	−0.084 [−0.226, 0.040]	0.820	0.92	1.030
Positive Affect Promotion Program	Intercept	1.091 [−2.511, 5.892]			
	EuroQoL VAS ^f^	−0.102 * [−0.256, −0.055]	0.844	0.903	0.966
	Positive Affect (PA-PANAS)	0.203 * [0.054, 0.557]	1.032	1.225	1.455

Notes: Healthy Lifestyle model: R^2^ = 0.25 (Hosmer & Lemeshow), R^2^ = 0.295 (Cox & Snell), R^2^ = 0.394 (Nagelkerke). X^2^_(1)_ = 16.11, *p* < 0.001; Mindfulness model: R^2^ = 0.005 (Hosmer & Lemeshow), R^2^ = 0.072 (Cox & Snell); R^2^ = 0.097 (Nagelkerke). X^2^_(2)_ = 3.422, *p* = 0.181; Positive Affect model: R^2^ = 0.29 (Hosmer & Lemeshow), R^2^ = 0.336 (Cox & Snell), R^2^ = 0.449 (Nagelkerke). X^2^_(2)_ = 16.358, *p* < 0.001; ^a^ Regression coefficient; ^b^ 95% bootstrap confidence intervals based on 1000 samples. ^c^ Confidence Interval; ^d^ Odds Ratio; ^e^ Positive dimension of the Positive and Negative Affect Schedule. ^f^ Visual Analog Scale of the EuroQoL. * *p* < 0.05.

## Data Availability

The data presented in this study are available on request from the corresponding author. The data are not publicly available due to legal and privacy issues.
